# Supplementary material for: Transcriptomic Profiling of Populus Roots Challenged with Fusarium Reveals Differential Responsive Patterns of Invertase and Invertase Inhibitor-Like Families within Carbohydrate Metabolism
Source: J Fungi (Basel). 2021 Jan 27;7(2):89. doi: 10.3390/jof7020089 (PMC7911864; doi:10.3390/jof7020089)
Supplement: Supplementary file 1 [file jof-07-00089-s001.zip › Supplementary Figure 1 and Table 1, 2 20201231.docx]

**Table S1.** Statistical list of reads mapping to the reference genome

| **Sample** | **FS0a** | **FS0b** | **FS0ac** | **FS24a** | **FS24b** | **FS24c** | **FS48a** | **FS48b** | **FS48c** | **FS72a** | **FS72b** | **FS72c** |
| --- | --- | --- | --- | --- | --- | --- | --- | --- | --- | --- | --- | --- |
| **Total reads** | 42393878 | 45029474 | 41418622 | 42734128 | 48460754 | 57493454 | 53957840 | 48869400 | 46513264 | 47448186 | 52288480 | 47450718 |
| **Total mapped** | 41110004(96.97%) | 43667301(96.97%) | 40056703(96.71%) | 40756299(95.37%) | 46177170(95.29%) | 54940391(95.56%) | 36476034(67.60%) | 32694586(66.90%) | 30878984(66.39%) | 29842999(62.90%) | 32961049(63.04%) | 30069609(63.37%) |
| **Multiple mapped** | 2051951(4.84%) | 2149024(4.77%) | 2899846(7.00%) | 2636737(6.17%) | 2554868(5.27%) | 2972152(5.17%) | 2112741(3.92%) | 1875534(3.84%) | 2709375(5.82%) | 1839729(3.88%) | 1986105(3.80%) | 1791453(3.78%) |
| **Uniquely mapped** | 39058053(92.13%) | 41518277(92.20%) | 37156857(89.71%) | 38119562(89.20%) | 43622302(90.02%) | 51968239(90.39%) | 34363293(63.69%) | 30819052(63.06%) | 28169609(60.56%) | 28003270(59.02%) | 30974944(59.24%) | 28278156(59.59%) |
| **Read-1** | 19545053(46.10%) | 20775667(46.14%) | 18593503(44.89%) | 19077533(44.64%) | 21821757(45.03%) | 25999787(45.22%) | 17191297(31.86%) | 15417477(31.55%) | 14089970(30.29%) | 14006477(29.52%) | 15493679(29.63%) | 14145670(29.81%) |
| **Read-2** | 19513000(46.03%) | 20742610(46.06%) | 18563354(44.82%) | 19042029(44.56%) | 21800545(44.99%) | 25968452(45.17%) | 17171996(31.82%) | 15401575(31.52%) | 14079639(30.27%) | 13996793(29.50%) | 15481265(29.61%) | 14132486(29.78%) |
| **Reads map '+'** | 19525354(46.06%) | 20754886(46.09%) | 18574763(44.85%) | 19056489(44.59%) | 21806174(45.00%) | 25979395(45.19%) | 17179534(31.84%) | 15407034(31.53%) | 14082545(30.28%) | 13999417(29.50%) | 15484578(29.61%) | 14136613(29.79%) |
| **Reads map '-'** | 19532699(46.07%) | 20763391(46.11%) | 18582094(44.86%) | 19063073(44.61%) | 21816128(45.02%) | 25988844(45.20%) | 17183759(31.85%) | 15412018(31.54%) | 14087064(30.29%) | 14003853(29.51%) | 15490366(29.62%) | 14141543(29.80%) |
| **Non-splice** | 23660101(55.81%) | 25112160(55.77%) | 22525435(54.38%) | 23017410(53.86%) | 26341902(54.36%) | 31363756(54.55%) | 21065229(39.04%) | 18888005(38.65%) | 17329953(37.26%) | 17311031(36.48%) | 19095621(36.52%) | 17463167(36.80%) |
| **Splice reads** | 15397952(36.32%) | 16406117(36.43%) | 14631422(35.33%) | 15102152(35.34%) | 17280400(35.66%) | 20604483(35.84%) | 13298064(24.65%) | 11931047(24.41%) | 10839656(23.30%) | 10692239(22.53%) | 11879323(22.72%) | 10814989(22.79%) |
| **Mapped in proper** | 37824350(89.22%) | 40235350(89.35%) | 35940722(86.77%) | 36974536(86.52%) | 42180128(87.04%) | 50324732(87.53%) | 33325594(61.76%) | 29830622(61.04%) | 27271674(58.63%) | 27073822(57.06%) | 29991192(57.36%) | 27387424(57.72%) |

^1^ FC, the fold change of normalized base mean value between two samples

**Table S2.** List of the DEGs based on the gene ontology (GO) classification at level 2^1^

| **Groups** | **Go Term** | **ID** | **FS24/FS0** | **FS48/FS0** | **FS72/FS0** |  |
| --- | --- | --- | --- | --- | --- | --- |
| Biological process  (BP) | Carbohydrate metabolism | GO:0005975 | 45 | 69 | 92 |  |
|  | Oxidation-reduction | GO:0055114 | 115 | 295 | 317 |  |
|  | Protein phosphorylation | GO:0006468 | 115 | 247 | 294 |  |
|  | Wounding response | GO:0009611 | 4 | 13 | 13 |  |
|  | Malate transport | GO:0015743 | 5 | 5 | 3 | |
|  | Protein ubiquitination | GO:0016567 | 14 | 19 | 27 | |
|  | Cell population proliferation | GO:0008283 | 3 | 6 | 7 | |
|  | Metal ion transport | GO:0030001 | 15 | 19 | 32 | |
|  | Defense response | GO:0006952 | 9 | 23 | 25 | |
|  | Oxidative stress response | GO:0006979 | 4 | 23 | 34 | |
|  | Nucleoside metabolism | GO:0009116 | 5 | 5 | 3 | |
|  | Chitin catabolism | GO:0006032 | 4 | 11 | 10 | |
|  | Regulation of transcription | GO:0006355 | 3 | 163 | 203 | |
|  | CW macromolecule catabolism | GO:0016998 | 3 | 11 | 10 | |
|  | Polysaccharide catabolism | GO:0000272 | n.a | 5 | 3 | |
| Cellular component (CC) | Membrane (M) | GO:0016020 | 74 | 115 | 157 | |
|  | Extracellular region | GO:0005576 | 3 | 6 | 7 | |
|  | M extrinsic component | GO:0019898 | 8 | 4 | 1 | |
|  | M integral component | GO:0016021 | 48 | 101 | 128 | |
|  | CW | GO:0005618 | 15 | 34 | 31 | |
|  | Extracellular matrix | GO:0031012 | 3 | 5 | 5 | |
|  | Exocyst | GO:0000145 | 4 | 5 | 5 | |
|  | Endoplasmic reticulum | GO:0005783 | n.a | 4 | 6 | |
|  | Kinesin complex | GO:0005871 | n.a | 3 | 10 | |
|  | Cytoplasm | GO:0005737 | n.a | 9 | 9 | |
|  | Nucleus | GO:0005634 | 2 | 32 | 47 | |
|  | Actin cytoskeleton | GO:0015629 | 1 | 2 | 3 | |
|  | Microtubule | GO:0005874 | 2 | 1 | 5 | |
| Molecular function (MF) | Ionotropic glutamate receptor | GO:0004970 | 18 | 17 | 22 | |
|  | FAD binding | GO:0050660 | 23 | 29 | 30 | |
|  | Transcription factor activity | GO:0003700 | 25 | 113 | 139 | |
|  | Specific DNA binding | GO:0043565 | 19 | 57 | 70 | |
|  | Heme binding | GO:0020037 | 19 | 104 | 109 | |
|  | Iron ion binding | GO:0005506 | 17 | 85 | 79 | |
|  | S-type endopeptidase inhibitor | GO:0004867 | 5 | 14 | 13 | |
|  | Hydrolase activity | GO:0004553 | 39 | 60 | 279 | |
|  | Peroxidase activity | GO:0004601 | 4 | 24 | 36 | |
|  | Polysaccharide binding | GO:0030247 | 19 | 23 | 30 | |
|  | Oxidoreductase activity-1 | GO:0016491 | 65 | 144 | 164 | |
|  | Oxidoreductase activity-2 | GO:0016705 | 15 | 79 | 73 | |
|  | Calcium ion binding | GO:0005509 | 18 | 17 | 21 | |
|  | Aspartic-type endopeptidase | GO:0004190 | 15 | 19 | 23 | |
|  | Protein kinase activity | GO:0004672 | 115 | 247 | 294 | |
|  | Calmodulin binding | GO:0005516 | 7 | 4 | 6 | |
|  | Growth factor | GO:0008083 | 3 | 6 | 7 | |
|  | Enzyme inhibitor activity | GO:0004857 | 4 | 29 | 24 | |
|  | Chitinase activity | GO:0004568 | 3 | 11 | 10 | |

^1^ the number of DEGs more than 2 annotated in the top 30 GO term. CW, cell wall; FAD, flavin adenine dinucleotide; n.a, not applicable


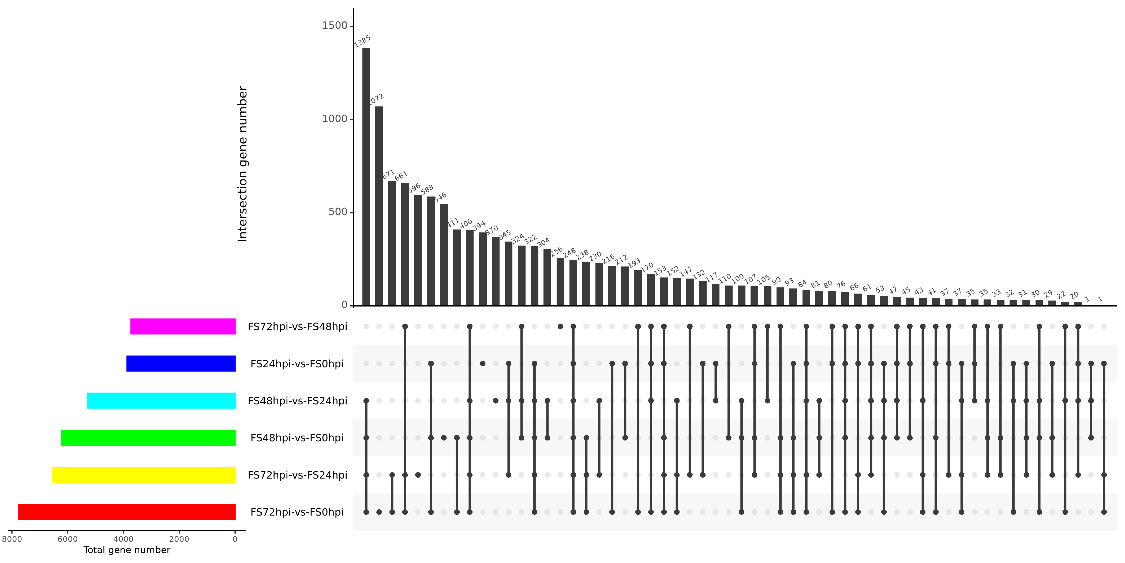


**Figure S1.** List of the annotated significantly expressed genes in six compared groups. The statistical of DEGs was based on the condition (FC was set up as 1.5, *p<0.01*).
