# Supplementary material for: Transcriptomic Profiling of Populus Roots Challenged with Fusarium Reveals Differential Responsive Patterns of Invertase and Invertase Inhibitor-Like Families within Carbohydrate Metabolism
Source: J Fungi (Basel). 2021 Jan 27;7(2):89. doi: 10.3390/jof7020089 (PMC7911864; doi:10.3390/jof7020089)
Supplement: Supplementary file 1 [file jof-07-00089-s001.zip › Supplementary Table 4 List of primers used for analyses of qRT-PCR 20201231.docx]

**Table S4.** List of primers used for analyses of qRT-PCR and fungal DNA quantification

| **Primer Name** | **Sequence** | **Access number/Gene name** | |
| --- | --- | --- | --- |
| **Plants** |  | |  |
| *β-Actin_qF* | GACCTTCAATGTGCCTGCAA | Potri.019G006700.1/*Ptβ-Actin* | |
| *β-Actin_qR* | ACCATCACCAGAATCCAGCA |  |  |
| *EF1𝛼_qF* | TCCGTCTTCCACTTCAGGATGTCT | Potri.006G130900.1/*PtEF1𝛼* | |
| *EF1𝛼_qR* | GTCACGACCATACCAGGCTTCAG |  |  |
| *UBIC_qF2* | TTGCTTGAGGAACTTGAAC | Potri.006G205700.7/*PtUBIC* | |
| *UBIC_qR2* | GTGAGGACCGATTACAGT |  |  |
| *qF-51* | TCACCAACAACACAGTGCTTC | Potri.001G108200.1 | |
| *qR-51* | AGTCACGTCACAGACCGTTT |  |  |
| *qF-91* | TAGAGGAGGTTGGGGACTCA | Potri.001G119300.1 | |
| *qR-91* | CAGCAAATCCATCCAAGCAGG |  |  |
| *qF-74* | CAGCGCCGTACAAAATAGCC | Potri.010G109300.1 | |
| *qR-74* | CGACCCGCGTATCTCATCAA |  |  |
| *qF-76* | CATGCATGTACAGATGTGGGC | Potri.016G001600.1 | |
| *qR-76* | AGCCCTCACAGTGATTGACC |  |  |
| *qF-80* | ATGCTCAGCCACTAGACCCT | Potri.003G113600.1 | |
| *qR-80* | CCTTGATGTCGAGTTGGTGC |  |  |
| *qF-10* | AGCCCTCACCAACAGGAAAG | Potri.003G123500.1 | |
| *qR-10* | CTGGTGAAGCAGGAGGATGG |  |  |
| *qF-17* | GCCACGGCCACATCATCTTA | Potri.007G108300.1 | |
| *qR-17* | TGGATAATCCGAGGCTCCCA |  |  |
| *qF-48* | TAGAGAGGCCACTGGCTGAT | Potri.008G132600.1 | |
| *qR-48* | ATGGTGATTCATCCTCGCCC |  |  |
| *qF-48* | TAGAGAGGCCACTGGCTGAT | Potri.008G102600.1/*PtC/VIF2* | |
| *qR-48* | ATGGTGATTCATCCTCGCCC |  |  |
| *qF-23* | TCCCGGAAGCTATTGAAGCC | Potri.010G063000.1/*PtC/VIF1* | |
| *qR-23* | GAGATTTGCCGTGGAAACCG |  |  |
| *qF-28* | CTGCCTTGTGTGTGCAATCC | Potri.012G127500.1 | |
| *qR-28* | ACAAGCCTGTCCACGGTATC |  |  |
| *qF-64* | GCCTAGACAAGGCTCAGTCC | Potri.015G128700.1 | |
| *qR-64* | AATCCTGGCCCTTGGATGAC |  |  |
| *qF-54* | GACGTCTAGCTCTCACTGCC | Potri.003G113700.1 | |
| *qR-54* | CAACAGCTCGACCCGTATGA |  |  |
| *qF-58* | GGCAGCCTAAAAGGTTCCGA | Potri.012G127400.1 | |
| *qR-58* | GTCAGCCTGGCAACATTCAC |  |  |
| *qF-92* | TCGGGCCTACATCGAAAACG | Potri.014G067500.1 | |
| *qR-92* | TCACGGATAAGGCAGCTTGG |  |  |
| *qF-42* | AGGAGCTCTTCCTTGGGAGT | Potri.006G210600.1/*PtCWI3* | |
| *qR-42* | CTCTACAGTGACGGTCTCGC |  |  |
| *qF-68* | TAGTGCAGTGGCCCATTGAG | Potri.006G227500.1/*PtCWI4* | |
| *qR-68* | AACCTCCACATCTGCCTGTG |  |  |
| *qF-44* | TGGTGTGGATCTGCGGTATG | Potri.006G227400.1/*PtCWI5* | |
| *qR-44* | GACTGAAGTCCAGACCAGCC |  |  |
| *qF-69* | CCCATCTCCGACGATCCAAG | Potri.003G112600.1/*PtVI2* | |
| *qR-69* | TCACGTTGGCCTTCTCTGAC |  |  |
| *qF-93* | TCCTTAGCAGATGCCACCAC | Potri.015G127100.1/*PtVI3* | |
| *qR-93* | TGAGCGCTTTGGTGCTTAGT |  |  |
| *qF-83* | TCAATGACCAGTGGGACGAA | Potri.013G006600.1/*PtCI2* | |
| *qR-83* | AGCAAATATGCACTGTATAGCCC |  |  |
| *qF-84* | AGCTTGGGGACACTCAACAG | Potri.008G024100.1/*PtCI3* | |
| *qR-84* | GCAGTAGCTCCCATGCTTCT |  |  |
| *qF-86* | TTACCAAACGGCCTGTGATTG | Potri.005G010800.1/*PtCI5* | |
| *qR-86* | TGGTGTTCAGAGAAGGAGGAGA |  |  |
| *qF-89* | TTCCATCTGTTCAATGCCGGA | Potri.004G167500.1/*PtCI9* | |
| *qR-89* | ACCACCAAATCAGGCTTCCC |  |  |
| *qF-90* | TTTCTGCAAAGACCATGCCC | Potri.013G110800.1/*PtCI12* | |
| *qR-90* | TTTCCAGTCCTTGATCGGCAA |  |  |
| ***F. solani*** |  |  | |
| *Fungal EF1-α F* | ATCGGCCACGTCGACTCT | MTCC-3871 | |
| *Fungal EF1-α R* | GGCGTCTGTTGATTGTTAGC |  |  |
